# Transmission-blocking compound candidates against *Plasmodium vivax* using *P. berghei* as an initial screening

**DOI:** 10.1590/0074-02760200513

**Published:** 2021-02-08

**Authors:** Camila Fabbri, Alexandre Oliveira Trindade, Francy’s Sayara Andrade, Macejane Ferreira de Souza, Claudia María Ríos-Velásquez, Marcus Vinicius Guimarães de Lacerda, Wuelton Marcelo Monteiro, Fabio Trindade Maranhão Costa, Rogerio Amino, Stefanie Costa Pinto Lopes

**Affiliations:** 1 Fundação de Medicina Tropical Dr Heitor Vieira Dourado, Instituto de Pesquisa Clínica Carlos Borborema, Manaus, AM, Brasil; 2 Universidade do Estado do Amazonas, Programa de Pós-Graduação em Medicina Tropical, Manaus, AM, Brasil; 3 Centro Universitário Fametro, Manaus, AM, Brasil; 4 Fundação Oswaldo Cruz-Fiocruz, Instituto Leônidas e Maria Deane, Manaus, AM, Brasil; 5 Universidade Estadual de Campinas, Campinas, SP, Brasil; 6 Institut Pasteur, Unit of Malaria Infection and Immunity, Department of Parasites and Insect Vectors, Paris, Île-de-France, France

**Keywords:** Malaria Box, membrane feeding assay and gametocytes

## Abstract

**BACKGROUND:**

Different strategies for improvement of malaria control and elimination are based on the blockage of malaria parasite transmission to the mosquito vector. These strategies include the drugs that target the plasmodial sexual stages in humans and the early developmental stages inside mosquitoes.

**OBJECTIVES:**

Here we tested Malaria Box compounds in order to evaluate their activity against male and female gametocytes in *Plasmodium berghei*, mosquito infection in *P. vivax* and ookinete formation in both species.

**METHODS/FINDINGS:**

The membrane feeding assay and the development of ookinetes by a 24 h *ex vivo* culture and the ookinete yield per 1000 erythrocytes were used to test transmission-blocking potential of the Malaria Box compounds in *P. vivax*. For *P. berghei* we used flow cytometry to evaluate male and female gametocyte time course and fluorescence microscopy to check the ookinete development. The two species used in this study showed similar results concerning the compounds’ activity against gametocytes and ookinetes, which were different from those in *P. falciparum.* In addition, from the eight Malaria Box compounds tested in both species, compounds MMV665830, MMV665878 and MMV665941 were selected as a hit compounds due the high inhibition observed.

**CONCLUSION:**

Our results showed that *P. berghei* is suitable as an initial screening system to test compounds against *P. vivax*.

Despite tremendous efforts, the global elimination of malaria is still a challenge for countries that have this endemic disease. *Anopheles* mosquitoes are responsible for the transmission and they became infected by ingesting *Plasmodium* gametocytes during a blood meal. These gametocytes undergo activation and a maturation process called gametogenesis and then disrupt the parasitophorous vacuole and the cell membrane to egress the host cell. Each male gametocyte forms eight microgametes by the exflagellation process, and then fertilises the macrogamete (female gamete) giving birth to a zygote. The zygote undergoes meiosis and originates motile ookinetes that crosses the mosquito’s midgut epithelial barrier to reach the basal lamina, where it develops an oocyst. After several multiplications, hundreds of sporozoites are formed and released to the hemolymph; thereafter they reach the salivary glands where it stays until being injected into another vertebrate host during a new mosquito blood-feeding. These five stages of *Plasmodium* spp. cycle are targets for the development of new compounds and strategies to control parasite transmission.^1^ ^,^ ^2^ ^,^ ^3^ ^,^ ^4^

Considering the unique features of *Plasmodium vivax* biology (e.g., lack of a long-term *in vitro* culture and the early appearance of gametocytes in the blood circulation) novel strategies for screening platforms of control strategies against this species are urged. Medicines for Malaria Venture (MMV), a not-for-profit public/private partnership organisation, created a box with 400 compounds with asexual antimalarial activity known for *P. falciparum*, namely ‘the Malaria Box’ (MB). Among these compounds, 200 have the potential to be developed as oral drugs. The other 200 are compounds that failed to be classified as potential oral drugs due to their physicochemical properties and are potential tools for probing biological mechanisms for malaria research.^3^ ^,^ ^4^ ^,^ ^5^ The MB compounds have been studied as potential transmission-blocking (T-B) drugs, i.e., gametocytocidal drugs that may decrease the infectious size of the reservoir by reducing gametocyte carriage.^1^ There is a need to look for compounds that target the activated and highly vulnerable gametocytes when they are undergoing gamete and ookinete formation in the mosquito midgut.^6^

The gold-standard methodology to assess the effectiveness of malaria T-B interventions *in vivo* is the membrane feeding assay (MFA), a methodology that estimates the infection (the presence of oocysts) and intensity (the quantity of oocysts) rates by dissecting the mosquito’s midgut and observing oocysts under light microscopy.^7^ Several studies in *P. falciparum* used this assay to validate new *in vitro* methodologies to test compounds’ efficacy, such as high-throughput assays, including the MB compounds.^8^ ^,^ ^9^ ^,^ ^10^ However, due to the considerable difference in biology between *P. falciparum* and *P. vivax*, compounds that show high activity in *P. falciparum* may not have the same effect on *P. vivax.* ^2^ Furthermore, there are no reports of MB compound studies in *P. vivax*, possibly a result of several factors: (i) the lack of a long-term *in vitro P. vivax* culture system, (ii) the absence of a high-throughput assay to test a large number of compounds for *P. vivax*, (iii) the restriction of access to samples to endemic areas, since culture is not a viable alternative, (iv) the expensive and limited access to animal models such as non-human primates, thus hampering experiments in non-endemic areas^11^ and, (v) *P. vivax* disease burden is usually underestimated and dwarfed by the enormous disease burden caused by *P. falciparum* in sub-Saharan Africa.^2^ However, *P. vivax* is the predominant parasite in the Region of the Americas. In Brazil, about 99% of malaria transmission are concentrated in Legal Amazon Region.^12^

Here MB compounds were tested as a T-B’s strategy for *P. vivax*. The direct membrane feeding assay methodology (DMFA) was used to check the ability of MB compounds to block the development of ookinetes *ex vivo*. We also investigated how the compounds specifically affected male and female gametocytes using a *P. berghei* rodent model.

## MATERIALS AND METHODS

*MB compounds and reagents* - To select the compounds to be tested in *P. vivax* T-B assays, since throughput assays are not feasible due to the lack of a continuous *in vitro* culture, we performed a literature review^8^ ^,^ ^9^ ^,^ ^10^ ^,^ ^13^ ^,^ ^14^ ^,^ ^15^ [Supplementary data (Table IV)] regarding MB compounds with known activity against *P. falciparum* gametocytes. Eight compounds were found reported to have cytotoxicity/inhibition above 50% to gametocytes *in vitro* or standard membrane feeding assay (SMFA) as follows: MMV000248, MMV006172, MMV019555, MMV019881, MMV665830, MMV665878, MMV665941 and MMV667491. The majority of these compounds are in probe-like subset, except MMV000248 and MMV665878, which are in drug-like subset. The compounds were obtained from MolPort^®^ and diluted in dimethyl sulphoxide (DMSO) at 2.5 or 5 mg/mL as a solution stock and maintaining at -80ºC. All reagents and medium were obtained from Sigma-Aldrich^©^.

*Mice and P. berghei infection* - *P. berghei* 820 cl1m1cl1, in which red fluorescent protein (RFP) is expressed by female gametocytes and green fluorescence protein (GFP) by male gametocytes^16^ were used to quantify male and female gametocytes by flow cytometry. *P. berghei* ANKA expressing GFP under the control of the hsp70 promoter^17^ were used for ookinete production. Four-week-old RjOrl:SWISS females (Elevage Janvier, France) were allocated to cages randomly with food (irradiated complete unique vegetable diet for rats, mice and hamsters Safe-diets) and water *ad libitum*. To infect mice, 100,000 to 150,000 parasites per microliter were inoculated by intraperitoneal injection. Parasites to be used in the *ex vivo/in vitro* assays were obtained by tail blood or cardiac puncture three days after infection. Parasitaemia and gametocytemia was checked by blood smear and/or by CytoFLEX S flow cytometer (Beckman Coulter).

*Compounds activity against male and female P. berghei gametocytes: time course* - To evaluate compound activity specifically against male and female *P. berghei* gametocytes, *P. berghei* 820 cl1m1cl1 parasites (final haematocrit of 2%, parasitaemia ranging from 5 to 9%) were incubated *ex vivo* with MB compounds diluted in 245 μL of gametocyte medium [RPMI1640 containing 25 mM HEPES, 20% foetal bovine serum (FBS) and 10 mM sodium bicarbonate at pH 7.6] in a final concentration of 10 μM in duplicates for a maximum of 24 h using a humidity chamber with 5% CO_2_ and 10% of O_2_ at 36ºC. Gametocytes viability was evaluated at 5 times points (1, 3, 6, 12 and 24 h) by flow cytometry - CytoFLEX S. Data were analysed by CytExpert 2.0 software.^18^

*Production of P. berghei ookinetes and drug assay* - To evaluate *P. berghei* male gametocyte exflagellation, 5 µL of infected tail blood were obtained and mixed immediately with 20 μL of ookinete medium (OokM) (RPMI1640 containing 25 mM HEPES, 20% FBS, 10 mM sodium bicarbonate and 50 mM xanthurenic acid at pH 7.6). After 10 min, exflagellation centres were counted in 12-15 fields using 63x objective fluorescence microscope (Zeiss Axio Observer). The same proportion of tail blood and OokM was used for ookinete formation. This solution was incubated for 2 h at 21ºC to allow completion of gametogenesis and fertilisation in a well plate.^19^ Then, each culture was diluted with 225 μL of OokM and MB compounds were added to a final concentration of 5 and 10 μM. The plate was incubated at 21ºC for a further 21-24 h to allow ookinete differentiation. Each compound was tested in four replicates. For ookinete measurement, 62.5 μL of OokM was removed of each well plate and the blood pellet was mixed with OokM remaining. 10 μL was added in a Glasstic slide 10 with grids (KOVA International) to count ookinetes in 27 fields using a 40x objective under fluorescence microscopy.

*P. vivax: infected patients* - Human participants were enrolled between October 2016 and May 2019. Patients diagnosed with *P. vivax* monoinfection by thick smears with parasitaemia higher than 1,000 parasites/µL in the absence of antimalarial treatment in the last 60 days were invited to participate in the study. The ones that agree to participate signed the informed consent and a sample of 10 mL of blood was withdrawn before treatment. All patients received antimalarial treatment as established in the Brazilian Malaria Guidelines.^20^

*Anopheles spp. colony* - *Anopheles aquasalis* females were reared at Laboratory of Medical Entomology at FMT-HVD in Manaus, Brazil. The colony was kept at a constant temperature (24-26ºC) and relative humidity (70-80%). Larvae were hatched in room temperature water and fed with fish food (TetraMin^®^). The larvae were allowed to pupate and emerge into adults in an enclosed mesh-covered cage with water and 10% sucrose available. Female *An. aquasalis* used for experiments were 3-6 days old.^21^

*P. vivax DMFA* - Blood samples collected in heparinised tubes were centrifuged to remove plasma. In a lamina flow chamber with a control temperature with 32-37ºC, erythrocytes were washed twice with RPMI-1640 and resuspended in inactivated human serum to 40% haematocrit. One millilitre of this solution was offered to three groups: control (with no compound included), 5 μM and 10 µM of each MB compound in groups of 120-150 female mosquitoes via membrane feeder devices at 37ºC until 2 h as previously described.^21^ The fully engorged mosquitoes were separated into different cages and kept until seven days post-feeding with available water and sucrose solution (10%). At day 7-post infection, mosquitoes were frozen; their midguts were dissected and stained with mercurochrome in order to determine infection rate [percentage of mosquitoes with one or more oocyst(s) in the midgut] and intensity (mean number of oocysts/dissected mosquitoes). The percentage drug inhibition of each compound was calculated by: [(mean of infection rate percentage control - mean of infection rate percentage MB compound) / mean of infection rate percentage control] x 100. For a consistent statistical analysis, only mosquitoes with at least one (1) oocyst in the midgut were considered in the infection intensity. Mosquitoes’ survival was monitored every day and mortality on day 7. The activity of each compound was evaluated in five different *P. vivax* patient isolates (biological replicates).

*Production of P. vivax ookinetes and drug assay* - The production of *P. vivax* ookinetes was evaluated as previously described,^22^ with few modifications. Briefly, parasites (haematocrit 20% and parasitaemia between 130-860 parasites per 200 leucocytes) were incubated for one hour in exflagellation medium at 24-25ºC. The resultant pellet of this incubation was resuspended in a 1:1 proportion with Roswell Park Memorial Institute (RPMI) 1640 medium, followed by a purification using with 45% percoll (percoll diluted in 50% of Milli-Q^®^ water and 5% of 10X RPMI 1640) in order to separate sexually mature stages of the parasite. Then, the resultant pellet was washed and resuspended in ookinete medium [Iscove Modified Dulbecco Media (IMDM) medium, 50 mg/litre of hypoxanthine and 20% heat-inactivated AB human serum, pH 8,0] to a 20% haematocrit. The MMV019881, MMV665941, MMV665830 and MMV665878 compounds were added at a final concentration of 10 μM and incubated at 24-25ºC for 24 h in order to evaluate the inhibition of zygotes to ookinetes development. The ookinete yield was determined per 1000 erythrocytes while percentage drug inhibition was calculated by: [(Control yield - MB compound yield) / Control yield] x 100. Three different *P. vivax* isolates (biological replicates) were evaluated for each compound.

*Statistical analysis* - Normality was evaluated by Shapiro-Wilk test. The comparison between two groups was performed by Mann Whitney test and multiple groups’. The comparison between three groups was performed by Kruskal-Wallis followed by Dunn’s multiple comparisons posttest. All correlations used the Spearman’s correlation coefficient (r). All analyses were performed using GraphPad Prism version 5 for Windows (GraphPad Software).

*Ethics* - All animal experiments were approved by the Animal Care and Use Committee of Institut Pasteur (CETEA Institut Pasteur 2013-0093, Ministère de l’Enseignement Supérieur et de la Recherche MESR 01324) and were performed in accordance with European guidelines and regulations (directive 2010/63/EU). All patients with *P. vivax* infection included in the project gave a written informed consent in protocols approved by FMT-HVD ethical board committee (CAAE: 50812815.0.0000.0005, approval number 1.358.054).

## RESULTS

*MB compounds have different activity in male and female P. berghei gametocytes* - Eight MB compounds identified in the literature [chemical properties are described in Supplementary data (Table I)] were chosen to be evaluated regarding their T-B activity against *P. berghei* and *P. vivax* malaria parasites.

Seven of the eight tested compounds showed activity against male and female *P. berghei* gametocytes after 24 h of exposure, with the exception of compound MMV019881 that did not show any activity against male and female gametocytes [Supplementary data (Table II)]. In male gametocytes the MB compounds MMV000248, MMV019555, MMV665830, MMV665878, MMV665941 and MMV667491 demonstrated activity start-up time after 6 h of exposure. In female gametocytes, MMV665830 showed activity after 1 h of exposure, MMV665941 after 3 h of exposure, MMV000248, MMV019555, MMV665878 and MMV667491 after 6 h of exposure.

**Fig. 1:**
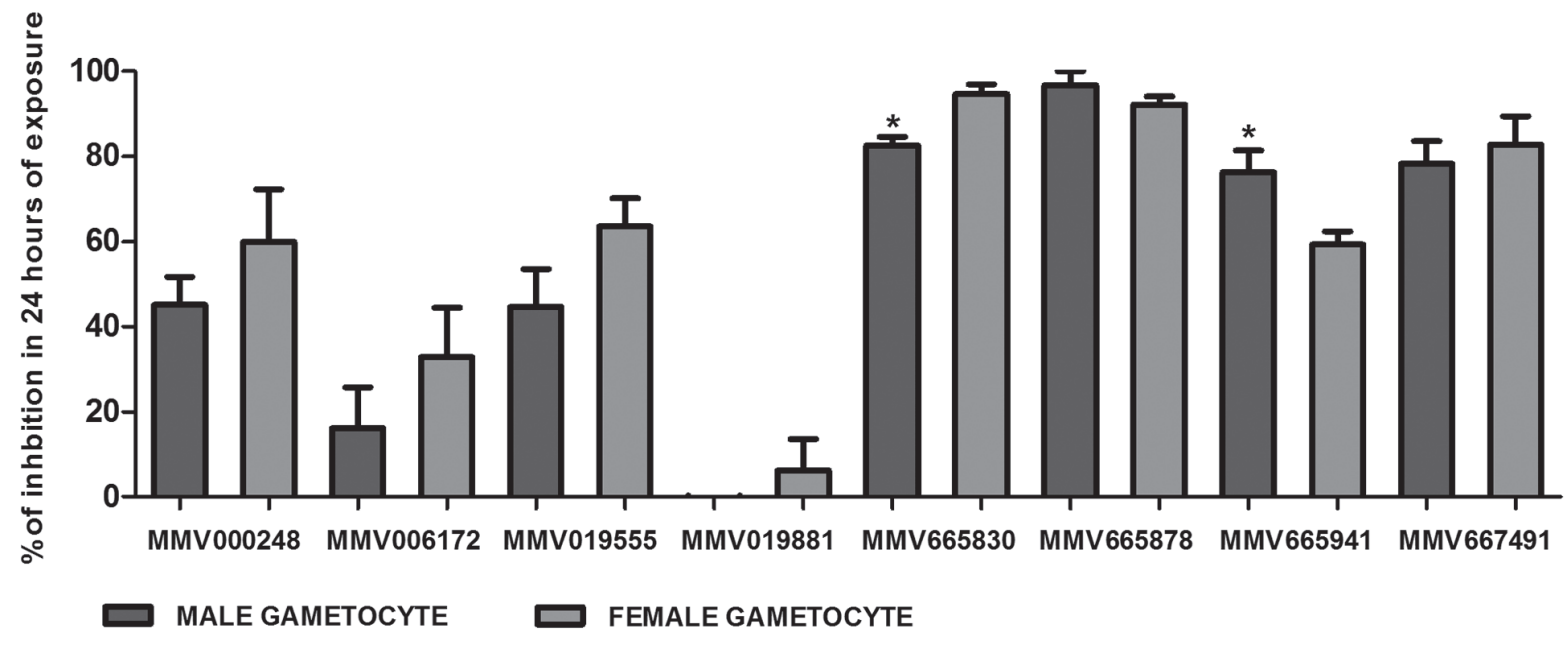
male and female *Plasmodium berghei* gametocytes cytotoxicity in 24 h of exposure to Malaria Box (MB) compounds. For difference between male and female gametocytes cytotoxicity in the same compound *p* values were calculated using Mann Whitney test and multiple groups’. Asterisks indicate p value < 0.05 for male and female gametocyte comparison per compound tested. Results are the average of two independent biological replicates.

Compound antimalarial activity at 10 µM against male and female *P. berghei* gametocytes was calculated at 24 h of exposure. Compounds MMV665830, MMV665878, MMV665941 and MMV667491 demonstrated cytotoxicity higher than 50% in both male and female gametocytes (Fig. 1). In contrast, the compounds MMV000248 (59.9%) and MMV019555 (63.6%) were more active against female gametocytes.

Interestingly, when the cytotoxicity percentage was compared between male and female gametocytes for the same compound, MMV665830 (p-value < 0.050) demonstrated higher activity in female gametocytes in comparison to MMV665941 (p-value < 0.050), which showed higher activity against male gametocytes.

*MB compounds are able to impair zygote to ookinete transformation in P. berghei* - To evaluate the capacity to block the zygote to ookinete transformation, the eight MB compounds were tested against *P. berghei* in an *ex vivo* ookinete culture (Table I). The compounds MMV019555, MMV665830, MMV665878 and MMV665941 showed a decrease in the number of ookinetes after 24 h of incubation when compared to the control group in both concentrations tested. Importantly, at the highest concentration, treatment with compounds MMV019555 and MMV665941 completely abrogated ookinete formation. Indeed, these compounds were effective even at the lowest concentration (5 μM) tested. Other compounds also showed high inhibition rates at both concentrations: MMV665830 and MMV665878.

**TABLE I:** *Plasmodium berghei* ookinete inhibition assay

	Ookinete (μL of blood)				Inhibition (%)	
MB compounds	Control	5 μM	Control	10 μM	5μ M	10 μM
MMV000248	8546.9 ± 3145.2	5718.8 ± 2144.1	7437.5 ± 3111.0	2843.8 ± 976.9	33.1	61.8
MMV006172	9703.1 ± 2216.0	4671.9 ± 2011.1	7906.3 ± 2188.4	2203.1 ± 1536.7	51.9	69.2
MMV019555	8546.9 ± 3145.2	1500.0 ± 1047.9^*b*^	7437.5 ± 3111.0	0 ± 0^*a*^	82.5	100.0
MMV019881	9703.1 ± 2216.0	7656.3 ± 3089.9	7906.3 ± 2188.4	5796.9 ± 2420.1	21.1	31.2
MMV665830	8546.9 ± 3145.2	458.3 ± 423.3^*a*^	7437.5 ± 3111.0	62.5 ± 104.6^*a*^	94.6	99.2
MMV665878	9703.1 ± 2216.0	1640.6 ± 1310.1^*b*^	7906.3 ± 2188.4	671.9 ± 353.2^*b*^	83.1	91.5
MMV665941	9703.1 ± 2216.0	1500 ± 923.4^*b*^	7906.3 ± 2188.4	0 ± 0^*a*^	84.5	100.0
MMV667491	8546.9 ± 3145.2	4890.6 ± 2165.5	7437.5 ± 3111.0	2312.5 ± 1095.9	42.8	68.9

Ookinete per microlitre of blood assay and inhibition rate in *P. berghei* GFP@Hsp70 using fluorescence microscope. P values were calculated using Kruskal-Wallis and Dunn’s multiple comparisons posttest. *a*: p value < 0.001; *b*: p value < 0.01 when compared to the control group. Results are the average of four independent biological replicates; MB: Malaria Box.

*MB compounds have activity against P. vivax sexual stages in vivo* - To evaluate the eight MB compounds activity in blocking the transmission of *P. vivax* to mosquitoes the gold standard DMFA was used in five independents assays with different isolates and a total of 7,482 *An. aquasalis* mosquitoes.

No compounds were able to reduce the infection rate significantly at the lowest concentration. However, there was an effect on infection intensity, with compounds MMV006172, MMV665941 and MMV667491 showing a decrease in the number of oocysts per midgut when compared to the control group (Table II).

**TABLE II:** *Plasmodium vivax* direct membrane feeding assay methodology (DMFA)

	Infection rate % (mosquitoes examined)			Infection intensity mean ± SD			Inhibition %	
MB compounds	Control	5 μM	10 μM	Control	5 μM	10 μM	5 μM	10 μM
MMV000248	80.0 (145)	54.3 (152)	37.9^*c*^ (152)	19.6 ± 18.7	16.1 ± 14.1	20.5 ± 16.9	32.2	52.6
MMV006172	82.9 (288)	50.7 (222)	35.5^*b*^ (216)	36.1 ± 32.9	6.1 ± 12.8^*a*^	6.3 ± 12.2^*a*^	38.9	57.2
MMV019555	74.9 (180)	77.1 (203)	53.2 (167)	25.5 ± 33.5	35.6 ± 43.9	25.5 ± 36.8	-2.9	29.0
MMV019881	76.5 (168)	75.3 (142)	61.3 (132)	22.6 ± 25.8	17.0 ± 19.1	13.2 ± 17.2	1.6	19.8
MMV665830	70.4 (165)	40.7 (179)	19.7^*b*^ (154)	3.9 ± 4.2	3.8 ± 5.9	2.5 ± 1.6	42.1	72.1
MMV665878	78.5 (254)	43.9 (193)	29.4^*b*^ (193)	28.7 ± 33.3	24.6 ± 32.3	30.9 ± 31.3	44.0	62.5
MMV665941	72.2 (234)	26.1 (206)	10.5^*b*^ (214)	13.1 ± 15.5	3.1 ± 2.5^*a*^	1.7 ± 1.0^*a*^	63.9	85.5
MMV667491	92.9 (190)	79.7 (240)	72.3^*c*^ (181)	38.4 ± 40.0	24.8 ± 36.2^*a*^	16.3 ± 21.3^*a*^	14.3	22.2

Infection rate indicates the presence or absence of oocyst per midgut, infection intensity was the mean of oocysts per midgut, and inhibition rate was calculated using the infection rate data of each compound compared to the control. P values were calculated using Kruskal-Wallis and Dunn’s multiple comparisons posttest. *a*: p value < 0.001; *b*: p value < 0.01; *c*: p value < 0.05 when compared to the control group. Results are the average of five independent biological replicates; MB: Malaria Box.

At the highest concentration the compounds MMV00248, MMV006172, MMV665830, MMV665878, MMV665941 and MMV667491 showed a decreased infection rate when compared to the control. Only the compounds MMV006172, MMV665941 and MMV667491 affected infection intensity, with a reduced the number of oocysts per midgut compared to the control (Table II). The compounds MMV019555 and MMV019881 did not show any significant activity concerning infection and intensity rate at both concentrations tested.

MMV665941 caused more than 50% inhibition at 5 μM and 10 μM concentrations, while MMV00248, MMV006172, MMV665830 and MMV665878 showed inhibition only at the highest concentration used. No MB compounds here evaluated showed any impact in mosquito mortality and survival when compared to the control group [Supplementary data (Table III, Fig. 1)].

*MB compounds have activity against zygote to ookinete transformation in P. vivax* - Compounds showing higher than 60% inhibition were considered as primary hits, with a second *ex vivo* methodology performed to confirm these findings. The compounds MMV665830, MMV665878 and MMV665941 (inhibition higher than 60% on DMFA methodology) and MMV019881 (negative control) were selected to evaluate the capacity to inhibit the zygote to ookinete transformation *ex vivo*.

After standardisation of the method, which was conducted using the methodology described,^22^ the four MB compounds were tested (Table III). The compounds MMV665830, MMV665878 and MMV665941 showed a reduction in ookinete development when compared with the control group that was higher than 60%. The low activity of MMV019881 compound showed by DMFA was confirmed with this methodology, with no statistic difference when compare to the control group.

Hence, the compounds MMV665830, MMV665878 and MMV665941 have presented an inhibitory tendency profile resembling the DMFA.

**TABLE III:** *Plasmodium vivax* ookinetes assay

	Ookinetes/10³ erythrocytes		Inhbition (%)
MB compounds	Control	10 μM	
MMV019881	37.7 ± 4.9	34.2 ± 6.2	9.3
MMV665830	72.3 ± 23.6	15.0 ± 2.8^*a*^	79.3
MMV665878	88.8 ± 17.7	3.83 ± 3.3^*a*^	91.5
MMV665941	70.0 ± 25.8	25.0 ± 8.4^*a*^	64.3

Ookinetes per 10³ erythrocytes assay in *P. vivax* using optical microscope. P values were calculated using Mann Whitney test and multiple groups’. *a*: p value < 0.01 when compared to the control group. Results are the average of three independent biological replicates; MB: Malaria Box.

## DISCUSSION

The eight MB compounds evaluated in this study demonstrated higher than 50% activity against gametocytes than the majority of *P. falciparum* studies available in the literature.^9^ ^,^ ^10^ ^,^ ^13^ ^,^ ^14^ ^,^ ^15^ ^,^ ^23^ However, here after T-B activity evaluation using two different parasite species (*P. berghei* and *P. vivax*) we found different results depending on the methodology and parasite stages evaluated. For example, the compound MMV019881, which demonstrated 88.2% of inhibition^10^ in a SMFA in *P. falciparum*, did not show any T-B activity in any assay performed in both species assessed in this study at the same concentration (10 μM). However, previous studies have demonstrated this compound’s activity against *P. berghei* in liver stage and in ookinetes.^4^ Previously, using a thermal shift assay a possible MMV019881 mechanism of action in *P. falciparum* was demonstrated involving the PfHSP90 protein,^4^ which is essential for parasite growth in human erythrocytes.

**Fig. 2:**
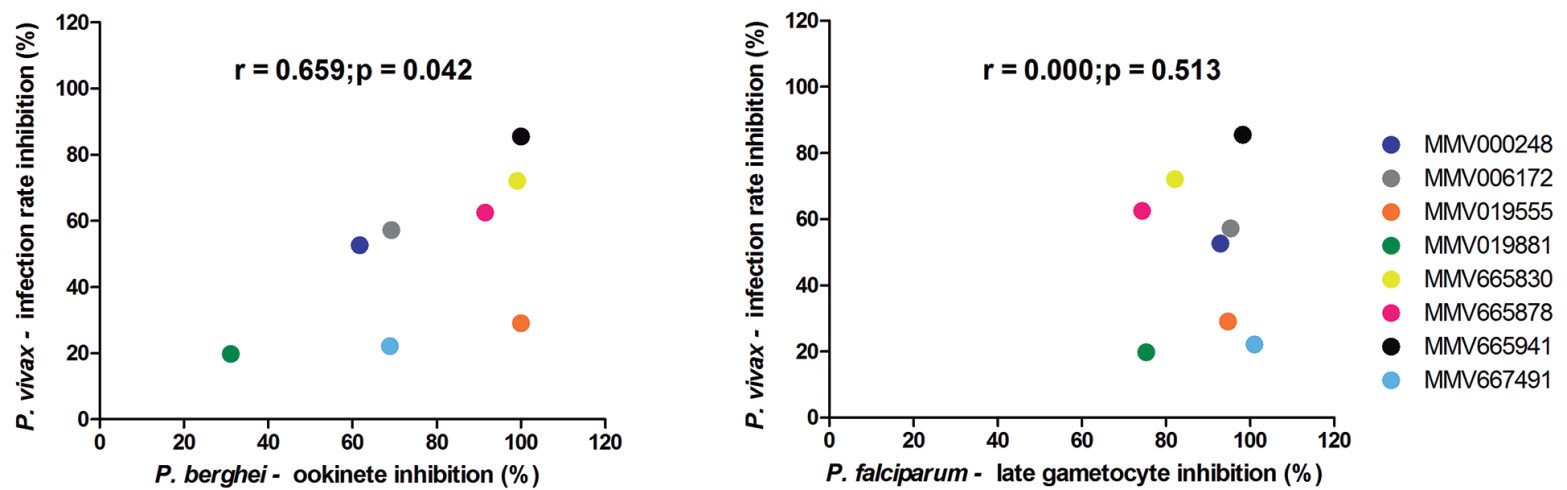
comparison of Malaria Box (MB) compounds transmission blocking activity between *Plasmodium vivax* and *P. berghei* or *P. falciparum.* (A) Spearman correlation between *P. berghei* ookinete inhibition - *ex vivo* (%) and infection rate in *P. vivax* for different MB compounds. (B) Spearman correlation between *P. falciparum* late gametocyte inhibition - *in vitro* (%) and infection rate in *P. vivax* for different MB compounds. The *P. falciparum* data were obtained from literature.^(8,13,14,24)^ Each coloured circle corresponds to one MB compound as indicated.

The compounds MMV019555 and MMV667491 exhibited contrasting results between *P. berghei* and *P. vivax* (Fig. 2)*.* MMV019555 impaired zygote to ookinete transformation in *P. berghei* (100% of inhibition) and showed activity start-up time of 6 h in male and female gametocytes presenting 63.6% cytotoxicity rate in female gametocytes at 24 h of incubation. In contrast, in *P. vivax* DMFA this compound did not reach 30% of infection rate inhibition, and thus we did not evaluate its effect on *P. vivax* zygote to ookinete transformation. The compound MMV667491 showed high inhibition in male and female *P. berghei* gametocytes, only moderately active against Pb ookinete and low inhibition in *P. vivax* DMFA*,* showing a slight decrease in infection rate and intensity at the two concentrations tested. Since *P. vivax* gametocytes are only incubated shortly with the drug during the DMFA (2 h maximum) this compound effect could be restricted to gametocyte stage and a longer incubation may be necessary to see its effect. Therefore this assay could miss gametocitocida’s activity once there is no previous incubation; and also since the fact that the compound is ingested by the mosquitoes, which takes into account the indirect antiparasitic effect, for example, the effect on mosquitoes microbiota and/or immune system that could favors or impairs *Plasmodium* sporogony.^21^

Interestingly, the compounds MMV000248 and MMV006172 (Fig. 2) showed similar activity in *P. berghei* ookinete transformation assay and *P. vivax* DMFA. However, MMV006172 demonstrated low inhibition in male and female *P. berghei* gametocytes.

All compounds mentioned above have been classified as hit compounds in *P. falciparum* T-B assays by two different studies impairing parasite transmission (inhibition of 100%) in assays using late gametocytes: MMV000248 and MMV019555;^13^ MMV006172 and MMV667491.^24^ Furthermore, the latter compound (MMV667491) may have an effect in *P. falciparum* phosphoethanolamine N-methyltransferase (PfPMT) activity.^4^ The physiological role of PfPMT seems to be in parasite membrane lipid biogenesis and in the development and survival of intraerythrocytic *P. falciparum*.^25^ Furthermore, this compound and others MMV compounds were tested against yeast growing in either fermentative or respiratory conditions.^4^ This effect could be non-specific in some cases (e.g., a growth-rate effect), but the results indicate that at least some MMV drugs are likely to have a respiratory target (the yeast *Saccharomyces cerevisiae* was used as a proxy for *P. falciparum*, because of killing similarities - the disruption of normal mitochondria function through membrane potential depolarisation).^26^

**Fig. 3:**
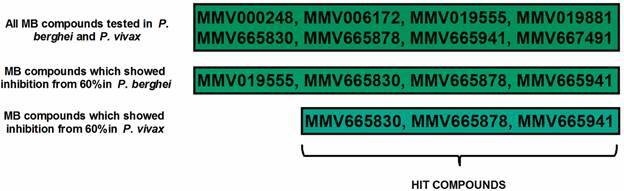
representative scheme showing the hit compounds. The three compounds MMV665830, MMV665878 and MMV665941 demonstrated high activity in both *Plasmodium* species.

The MB compounds MMV665830, MMV665878 and MMV665941 exhibited inhibition higher than 60% in all assays and in both species tested (Figs 2, 3). Hence, these three compounds were classified as hit compounds in this study. Indeed, MMV665830 reached the higher inhibition in female gametocytes and in *P. berghei* ookinete transformation. In *P. vivax*, this compound showed the second-best inhibition rate in DMFA and in ookinete inhibition.

The MMV665941 showed a higher inhibition against male gametocytes in comparison to female gametocytes, whereas MMV665830 showed the opposite profile. In addition, the compound MMV665941 was able to block the zygote to ookinete transformation (100% of inhibition) in *P. berghei*, showed the highest inhibition in infection rate and decreased infection intensity in both concentrations tested in *P. vivax* DMFA. In ookinete transformation, MMV665941 showed the third best inhibition rate. This compound, which is also called methylrosaniline, has a dermatological topical use,^27^ and may target a sterol-dependent function. *Plasmodium* parasites require sterol lipids, which they can scavenge from the host.^28^ In a previous study, out of seven MMV665941-sensitive yeast mutants to this compound, two were defective for sterol synthesis (*erg2Δ* and *erg6Δ*) revealing the importance of this metabolic pathway for this compound’s action.^4^

Lastly, MMV665878 showed an inhibition higher than 90% in *P. berghei*. In *P. vivax*, this compound exhibited the third best DMFA inhibition rate and the best inhibition in ookinete rate. Two possible mechanisms of action are described in the literature for this compound. MMV665878 inhibits PfATP4 activity - a membrane plasmatic protein which functions as a sodium/potassium pump - increasing the sodium concentration and the pH of the cytosol.^29^ The second mechanism is related to a possible respiratory target.^4^

Limitations of this study were the result of *P. vivax* assay. It was not possible to evaluate a large number of MB compounds in *P. vivax* due mostly to the absence of an *in vitro* long-term culture. The MFA methodology, the gold standard assay for T-B drug activity evaluation, requires laborious dissection of anophelines’ midgut and thus did not allow studying several compounds. Moreover, there are no positive control drugs described in literature until now, which therefore limit the standardisation of this assay. Our research group has already studied the effect of ivermectin in T-B,^30^ demonstrating that only the drug’s metabolite is highly effective, which makes its use as a control drug in this methodology unfeasible.

However, the *P. vivax* ookinete methodology presented here may offer an alternative to evaluating T-B activity in studies with a large number of compounds, since it has demonstrated an inhibitory tendency profile resembling the DMFA in all four compounds herein evaluated, including the useful addition of MMV019881 as a negative control. The methodology could be used as a primary screening followed by the gold standard method in order to avoid the hard work on mosquitoes’ dissection.

In spite of the limitations mentioned above, the eight MB compounds tested here demonstrated that *P. berghei* is suitable as an initial screening for testing compounds against *P. vivax.* Indeed, we found a correlation between *P. berghei* and *P. vivax* T-B data; which we have not encountered for *P. falciparum* and *P. vivax* (Fig. 2) and for *P. falciparum* and *P. berghei* [Supplementary data (Fig. 2)].

The literature show that *P. falciparum* remains a good approach for screening a large number of compounds,^8^ ^,^ ^9^ ^,^ ^10^ ^,^ ^24^ but due to differences in parasite biology between *P. vivax* and other species, this screening may miss some potential candidates or show false positives that must then be validated in *P. vivax.* In contrast, *P. berghei* seems to be even more promising as a screening for *P. vivax* demonstrating a very similar phenotype.

Here similarities are demonstrated concerning *P. vivax.* and *P. berghei* cytotoxicity and inhibition of conversion rates of gametocytes and ookinetes for three MMV compounds with high levels of inhibition. *P. berghei* proved an alternative for initial screening focusing on compounds with T-B activity against *P. vivax*.

## References

[1] Gonçalves D, Hunziker P (2016). Transmission-blocking strategies the roadmap from laboratory bench to the community. Malar J.

[2] Mueller I, Galinski MR, Baird JK, Carlton JM, Kochar DK, Alonso PL (2009). Key gaps in the knowledge of Plasmodium vivax, a neglected human malaria parasite. Lancet Infect Dis.

[3] Spangenberg T, Burrows JN, Kowalczyk P, McDonald S, Wells TNC, Willis P (2013). The open access malaria box a drug discovery catalyst for neglected diseases. PLoS One.

[4] Van Voorhis WC, Adams JH, Adelfio R, Ahyong V, Akabas MH, Alano P (2016). Open source drug discovery with the malaria box compound collection for neglected diseases and beyond. PLoS Pathog.

[5] MMV Switzerland: medicines for malaria venture. http://www.mmv.org/.

[6] Sinden RE (2017). Developing transmission-blocking strategies for malaria control. PLoS Pathog.

[7] Churcher TS, Blagborough AM, Delves M, Ramakrishnan C, Kapulu MC, Williams AR (2012). Measuring the blockade of malaria transmission - An analysis of the standard membrane feeding assay. Int J Parasitol.

[8] Ruecker A, Mathias DK, Straschil U, Churcher TS, Dinglasan RR, Leroy D (2014). A male and female gametocyte functional viability assay to identify biologically relevant malaria transmission-blocking drugs. Antimicrob Agents Chemother.

[9] Plouffe DM, Wree M, Du AY, Meister S, Li F, Patra K (2016). High-throughput assay and discovery of small molecules that interrupt malaria transmission. Cell Host Microbe.

[10] Vos MW, Stone WJR, Koolen KM, Van Gemert G-J.Van Schaijk B.Leroy D (2015). A semi-automated luminescence based standard membrane feeding assay identifies novel small molecules that inhibit transmission of malaria parasites by mosquitoes. Sci Rep.

[11] Galinski MR, Meyer EVS, Barnwell JW (2013). Plasmodium vivax Modern strategies to study a persistent parasite's life cycle. In Advances in parasitology. Elsevier.

[12] WHO (2019). World malaria report. https://www.who.int/publications/i/item/world-malaria-report-2019.

[13] Bowman JD, Merino EF, Brooks CF, Striepen B, Carlier PR, Cassera MB (2014). Antiapicoplast and gametocytocidal screening to identify the mechanisms of action of compounds within the malaria box. Antimicrob Agents Chemother.

[14] Sanders NG, Sullivan DJ, Mlambo G, Dimopoulos G, Tripathi AK (2014). Gametocytocidal screen identifies novel chemical classes with Plasmodium falciparum transmission blocking activity. PLoS One.

[15] Lucantoni L, Silvestrini F, Signore M, Siciliano G, Eldering M, Dechering KJ (2015). A simple and predictive phenotypic high content imaging assay for Plasmodium falciparum mature gametocytes to identify malaria transmission blocking compounds. Sci Rep.

[16] Ponzi M, Sidén-kiamos I, Bertuccini L, Currà C, Kroeze H, Camarda G (2009). Egress of Plasmodium berghei gametes from their host erythrocyte is mediated by the MDV-1 / PEG3 protein. Cell Microbiol.

[17] Ishino T, Orito Y, Chinzei Y, Yuda M (2006). A calcium-dependent protein kinase regulates Plasmodium ookinete access to the midgut epithelial cell. Mol Microbiol.

[18] Boisson B, Lacroix C, Bischoff E, Gueirard P, Bargieri DY, Franke-fayard B (2011). The novel putative transporter NPT1 plays a critical role in early stages of Plasmodium berghei sexual development. Mol Microbiol.

[19] Guttery DS, Poulin B, Ferguson DJP, Wickstead B, Carroll PL, Ramakrishnan C (2012). A unique Protein Phosphatase with Kelch-Like Domains (PPKL) in Plasmodium modulates ookinete differentiation, motility and invasion. PLoS Pathog.

[20] MS (2019). Guia de tratamento da malária no Brasil. https://www.saude.gov.br/bvs.

[21] Pimenta PFP, Orfano AS, Bahia AC, Duarte APM, Ríos-Velásquez CM, Melo FF (2015). An overview of malaria transmission from the perspective of Amazon Anopheles vectors. Mem Inst Oswaldo Cruz.

[22] Bounkeua V, Li F, Chuquiyauri R, Abeles SR, Mcclean CM, Neyra V (2011). Lack of molecular correlates of Plasmodium vivax ookinete development. Am J Trop Med Hyg.

[23] Lucantoni L, Avery V (2012). Whole-cell in vitro screening for gametocytocidal compounds. Future Med Chem.

[24] Duffy S, Avery VM (2013). Identification of inhibitors of Plasmodium falciparum gametocyte development. Malar J.

[25] Witola WH, Bissati KEl, Pessi G, Xie C, Roepe PD, Mamoun CB (2008). Disruption of the Plasmodium falciparum PfPMT gene results in a complete loss of phosphatidylcholine biosynthesis via the serine-decarboxylase-phosphoethanolamine-methyltransferase pathway and severe growth and survival defects* J Biol. Chem.

[26] Wang J, Huang L, Li J, Fan Q, Long Y, Li Y (2010). Artemisinin directly targets malarial mitochondria through its specific mitochondrial activation. PLoS One.

[27] Gloor M, Wolnicki D (2001). Anti-irritative effect of methylrosaniline chloride (gentian violet). Dermatology.

[28] Coppens I (2013). Targeting lipid biosynthesis and salvage in apicomplexan parasites for improved chemotherapies. Nat Rev Microbiol.

[29] Lehane AM, Ridgway MC, Baker E, Kirk K (2014). Diverse chemotypes disrupt ion homeostasis in the malaria parasite. Mol Microbiol.

[30] Pinilla YT, Lopes SCP, Sampaio VS, Andrade FS, Melo GC, Orfanó AS (2018). Promising approach to reducing malaria transmission by ivermectin sporontocidal effect against Plasmodium vivax in the South American vectors Anopheles aquasalis and Anopheles darlingi. PLoS Negl Trop Dis.

