# Supplementary material for: Transmission-blocking compound candidates against Plasmodium vivax using P. berghei as an initial screening
Source: Mem Inst Oswaldo Cruz. 2021 Feb 8;116:e200513. doi: 10.1590/0074-02760200513 (PMC7874845; doi:10.1590/0074-02760200513)
Supplement: Supplementary file 1 [file 1678-8060-mioc-116-e200513-s.pdf]

TABLE I
Malaria Box (MB) compounds

Compounds	CHEMBLID	Structure	Chemical properties
MMV000248	CHEMBL394283	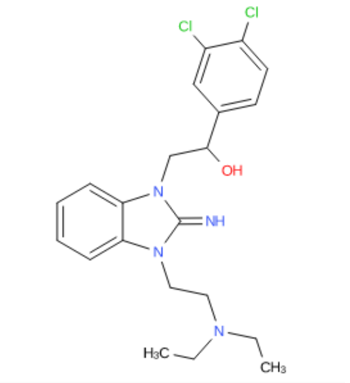	Name: no data<br>Molecular species: BASE<br>Molecular weight: 421.37 g/mol<br>Molecular formula: C21H26Cl2N4O
MMV006172	CHEMBL106525	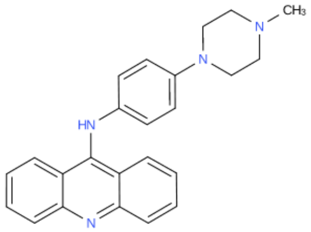	Name: no data<br>Molecular species: BASE<br>Molecular weight: 368.48 g/mol<br>Molecular formula: C24H24N4
MMV019555	CHEMBL73800	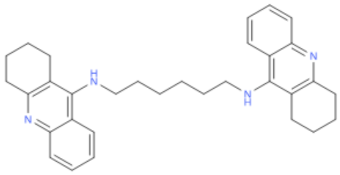	Name: no data<br>Molecular species: BASE<br>Molecular weight: 478.68 g/mol<br>Molecular formula: C32H38N4
MMV019881	CHEMBL546799	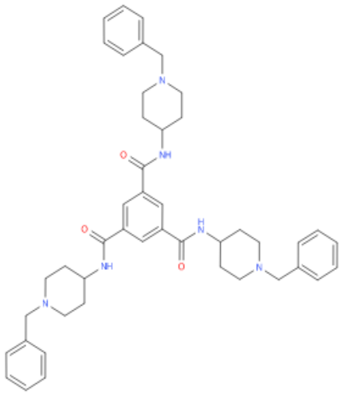	Name: no data<br>Molecular species: NEUTRAL<br>Molecular weight: 726.97 g/mol<br>Molecular formula: C45H54N6O3
MMV665830	CHEMBL602580	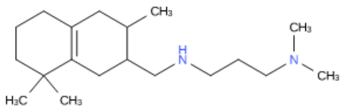	Name: no data<br>Molecular species: BASE<br>Molecular weight: 292.51 g/mol<br>Molecular formula: C19H36N2

Compounds	CHEMBLID	Structure	Chemical properties
MMV665878	CHEMBL530223	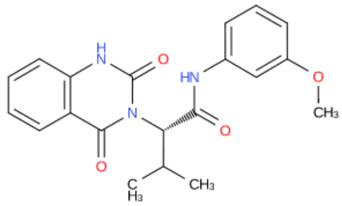	Name: no data<br>Molecular species: NEUTRAL<br>Molecular weight: 367.41 g/mol<br>Molecular formula: C20H21N3O4
MMV665941	CHEMBL592105	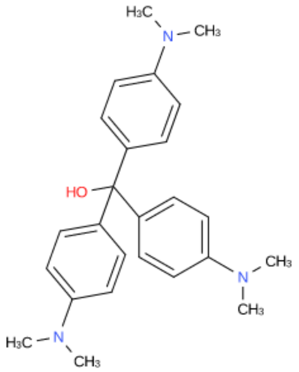	Name: METHYLROSANILINE<br>Molecular species: NEUTRAL<br>Molecular weight: 389.54 g/mol<br>Molecular formula: C25H31N3O
MMV667491	CHEMBL1437888	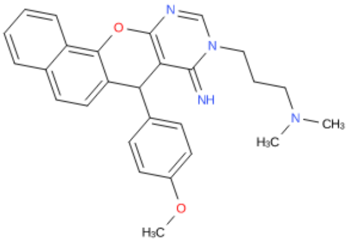	Name: no data<br>Molecular species: BASE<br>Molecular weight: 440.55 g/mol<br>Molecular formula: C27H28N4O2

MB compounds' chemical properties. The table shows the CHEMBLID name, the chemical structure, molecular species, molecular weight, molecular formula of the eight compounds tested in this study.

TABLE II
Male and female *Plasmodium berghei* gametocytes

	Activity start-up time		Inhibition after 24 h of exposure	
MB compounds	♂ gametocyte (%)	♀ gametocyte (%)	♂ gametocyte (%)	♀ gametocyte (%)
MMV000248	6 h[a]	6 h[a]	45.2	59.9
MMV006172	24 h[c]	12 h[b]	16.2	32.9
MMV019555	6 h[a]	6 h[a]	40.6	63.6
MMV019881	no activity	no activity	(-)14.6	6.2
MMV665830	6 h[a]	1 h[a]	82.5[c]	94.6
MMV665878	6 h[b]	6 h[a]	96.7	92.1
MMV665941	6 h[a]	3 h[a]	76.3[c]	59.3
MMV667491	6 h[a]	6 h[a]	78.3	82.8

Male and female activity start-up time (the first hour which the compound had activity compared to the control group) and inhibition rate in *P. berghei* 820 cl1m1cl1 strain. P values were calculated using 2way ANOVA with Bonferroni posttests. *a*: p value < 0.001; *b*: p value < 0.01; *c*: p value < 0.05 when compared to the control group. Results are the average of two independent biological replicates; MB: Malaria Box.

TABLE III

Anopheles aquasalis mortality

MB compounds	Control (%)	5 μM (%)	10 μM (%)
MMV000248	38.5	36.4	33.4
MMV006172	26.3	24.8	24.0
MMV019555	23.2	23.4	27.1
MMV019881	34.4	30.9	35.1
MMV665830	21.0	23.2	24.7
MMV665878	24.0	32.2	30.3
MMV665941	50.1	42.0	34.0
MMV667491	14.6	19.6	20.3

The *An. aquasalis* mortality after seven day of Malaria Box (MB) compounds exposure. P values were calculated using Kruskal-Wallis and Dunn's multiple comparisons posttest. No differences between groups was found. Data are the mean of five independent biological replicates.

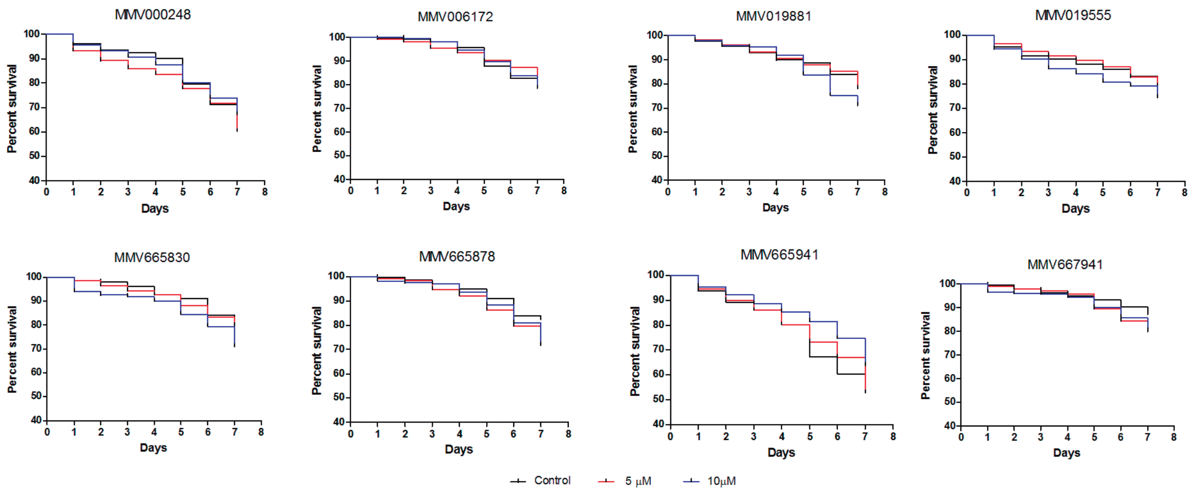

Fig. 1: Malaria Box compounds' survival graphics. Results are the average of five independent biological replicates. The comparison of survival curves by Mantel-Cox test did not show differences between the control versus 5 μM and 10 μM groups.

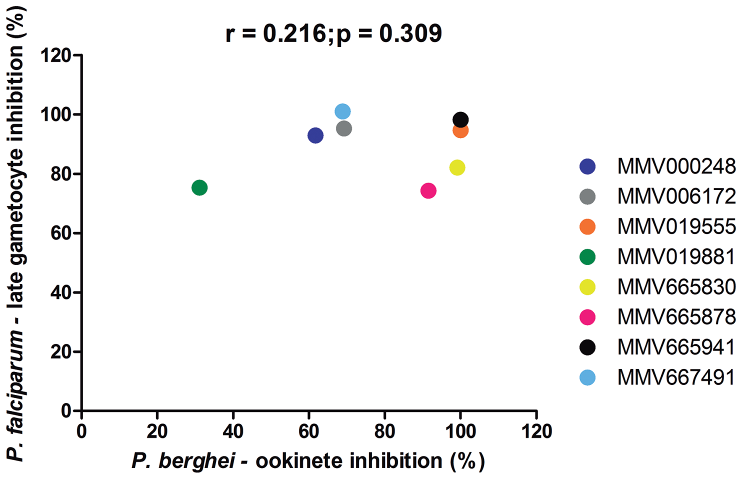

Fig. 2: spearman correlation between *Plasmodium falciparum* late gametocyte inhibition - *in vitro* (%) and *P. berghei* ookinete inhibition - *ex vivo* (%) for different Malaria Box (MB) compounds. The *P. falciparum* data were obtained from literature.[8,13,14,24] Each coloured circle corresponds to one MB compound as indicated.

TABLE IV

		Plasmodium falciparum												
		SMFA			gametocyte late stage - inhibition %			gametocyte late stage - IC50 (nM)				female gamete inh %		male gamete inh %
COMPOUNDS	CHEMBLID	(direct) % inhibition [10µM][1]	% inhibition [1µM][2]	IC50 (nM)[3]	NF54-pfs16 GFP-luc [5µM][4]	SYBR Green % [10µM][6]	luciferase [12.5µM][3]	luciferase assay[7]	fluorescence NF54- 1[5]	Fluorescence[8]	SYBR Green[6]	microscope and imaging [1µM][2]	3D7A acridine orange % [5µM][9]	microscope and imaging [1µM][2]
MMV665878	CHEMBL530223	-	-	-	59,9	98,9	65,1	-	-	2635	1100	36,6	77,1	70,9
MMV006172	CHEMBL106525	-	-	-	104,3	102,9	87,1	713,8	420	1482	2600	-3,1	105,6	56,4
MMV665941	CHEMBL592105	99,8	-	1040	102,6	116,3	88,2	885,1	-	5899	1800	44,6	91,5	19,7
MMV019555	CHEMBL73800	-	-	-	86,9	97,7	94,0	-	470	4686	3400	-9,4	60,7	18,8
MMV019881	CHEMBL546799	88,2	-	1170	31,9	95,2	97,9	752,5	-	2957	5500	-6,9	41,5	57,0
MMV665830	CHEMBL602580	-	-	-	85,3	97,2	68,0	811,5	-	1663	3300	56,1	70,3	62,3
MMV000248	CHEMBL394283	48,8	-	1660	89,4	95,2	87,1	605,6	310	1482	-	8,0	49,4	-66,0
MMV667491	CHEMBL1437888	89,1	96,9	60	114,7	98,6	99,2	880,8	-	2635	4500	27,3	78,8	83,6

REFERENCES

1. Vos MW, Stone WJR, Koolen KM, van Gemert G-J, van Schaijk B, Leroy D, et al. A semi-automated luminescence based standard membrane feeding assay identifies novel small molecules that inhibit transmission of malaria parasites by mosquitoes. Sci Rep. 2015; 5: 1-13.

2. Ruecker A, Mathias DK, Straschil U, Churcher TS, Dinglasan RR, Leroy D, et al. A male and female gametocyte functional viability assay to identify biologically relevant malaria transmission-blocking drugs. Antimicrob Agents Chemother. 2014; 58: 7292-302.

3. Plouffe DM, Wree M, Du AY, Meister S, Li F, Patra K, et al. High-throughput assay and discovery of small molecules that interrupt malaria transmission. Cell Host Microbe. 2016; 19: 114-26.

4. Duffy S, Avery VM. Identification of inhibitors of *Plasmodium falciparum* gametocyte development. Malar J. 2013; 12: 1-15.

5. Bowman JD, Merino EF, Brooks CF, Striepen B, Carlier PR, Cassera MB. Antiapicoplast and gametocytocidal screening to identify the mechanisms of action of compounds within the Malaria Box. Antimicrob Agents Chemother. 2014; 58: 811-9.

6. Sanders NG, Sullivan DJ, Mlambo G, Dimopoulos G, Tripathi AK. Gametocytocidal screen identifies novel chemical classes with *Plasmodium falciparum* transmission blocking activity. PLoS One. 2014; 9: 1-13.

7. D'Alessandro S, Camarda G, Corbett Y, Siciliano G, Parapini S, Cevenini L, et al. A chemical susceptibility profile of the *Plasmodium falciparum* transmission stages by complementary cell-based gametocyte assays. J Antimicrob Chemother. 2016; 71: 1148-58.

8. Sun W, Tanaka TQ, Magle CT, Huang W, Southall N, Huang R, et al. Chemical signatures and new drug targets for gametocytocidal drug development. Sci Rep. 2014; 4: 1-11.

9. Lucantoni L, Silvestrini F, Signore M, Siciliano G, Eldering M, Dechering KJ, et al. A simple and predictive phenotypic high content imaging assay for *Plasmodium falciparum* mature gametocytes to identify malaria transmission blocking compounds. Sci Rep. 2015; 5: 1-14.
